# Feasibility and Findings of Including Self-Identified Adult Congenital Heart Disease Patients in the INVESTED Trial

**DOI:** 10.1016/j.jacadv.2024.100897

**Published:** 2024-03-14

**Authors:** Payam Dehghani, Varun Srivatsav, Orly Vardeny, Jasmine Grewal, Alexander R. Opotowsky, Isabelle Vonder Muhll, Michelle Keir, Robin Ducas, Jyotpal Singh, KyungMann Kim, Jacob Joseph, Jamil Aboulhosn, Tom Havighurst, Sheila M. Hegde, Deepak L. Bhatt, Scott Solomon, Michael Farkouh, Shaun G. Goodman, Tabitha G. Moe, Jacob A. Udell

**Affiliations:** a Division of Cardiology, Department of Medicine, Prairie Vascular Research Inc, Regina, Saskatchewan, Canada; b Department of Medicine, University of Saskatchewan, Saskatoon, Saskatchewan, Canada; c Minneapolis VA Center for Care Delivery and Outcomes Research, University of Minnesota, Minneapolis, Minnesota, USA; d Division of Cardiology, Department of Medicine, University of British Columbia, Vancouver, British Columbia, Canada; e Department of Pediatrics, Heart Institute, Cincinnati Children's Hospital, University of Cincinnati College of Medicine, Cincinnati, Ohio, USA; f Division of Cardiology, University of Alberta, Edmonton, Alberta, Canada; g Libin Cardiovascular Institute, University of Calgary, Calgary, Alberta, Canada; h Section of Cardiology, Department of Internal Medicine, University of Manitoba, Winnipeg, Manitoba, Canada; i Department of Biostatistics and Medical Informatics, University of Wisconsin-Madison, Madison, Wisconsin, USA; j Cardiology Section, VA Providence Healthcare System, Providence, Rhode Island, USA; k Ahmanson/UCLA Adult Congenital Heart Center, Ronald Reagan/UCLA Medical Center, Los Angeles, California, USA; l Division of Cardiovascular Medicine, Brigham and Women's Hospital, Boston, Massachusetts, USA; m Mount Sinai Heart, Icahn School of Medicine at Mount Sinai Health System, New York, New York, USA; n Academic Affairs, Cedars-Sinai Health System, Los Angeles, California, USA; o Division of Cardiology, St. Michael's Hospital, Unity Health Toronto, Peter Munk Cardiac Centre, University Health Network, University of Toronto, Toronto, Ontario, Canada; p Canadian VIGOUR Centre, University of Alberta, Edmonton, Alberta, Canada; q Arizona Cardiology Group, University of Arizona College of Medicine-Phoenix, Phoenix, Arizona, USA; r Faculty of Medicine, Division of Cardiology, Department of Medicine, Women’s College Hospital, University of Toronto, Toronto, Ontario, Canada

**Keywords:** adult congenital heart disease, influenza, vaccination

## Abstract

**Background:**

Adult congenital heart disease (ACHD) patients have significant morbidity and rise in cardiac admissions. Their outcome with high-dose influenza vaccination is unknown in comparison to those without ACHD.

**Objectives:**

The purpose of this study was to compare all-cause mortality or cardiopulmonary hospitalizations in self-identified ACHD versus non-ACHD patients receiving high- or low-dose influenza vaccination within the INfluenza Vaccine to Effectively Stop cardioThoracic Events and Decompensated heart failure trial.

**Methods:**

We prospectively included ACHD patients in the INVESTED (INfluenza Vaccine to Effectively Stop cardioThoracic Events and Decompensated heart failure) trial. The primary endpoint was all-cause death or hospitalization for cardiovascular or pulmonary causes.

**Results:**

Of the 272 ACHD patients, 132 were randomly assigned to receive high-dose trivalent and 140 to standard-dose quadrivalent influenza vaccine. Compared to the non-ACHD cohort (n = 4,988), ACHD patients were more likely to be younger, women, smokers, have atrial fibrillation, and have a qualifying event of heart failure. The primary outcome was 49.8 events versus 42.8 events per 100 person-years (adjusted HR: 1.17; 95% CI: 0.95-1.45; *P* = 0.144) in the ACHD group and non-ACHD group, respectively. The interaction between ACHD status and randomized treatment effect was not significant for the primary outcome (*P* = 0.858). Vaccine-related adverse events were similar in both groups.

**Conclusions:**

Patients who self-identify as being ACHD had similar primary outcome of all-cause death or hospitalization for cardiovascular or pulmonary causes compared to non-ACHD cohort. High-dose influenza vaccination was similar to standard-dose influenza vaccination on the primary outcome in patients who self-identify as ACHD.

Influenza causes morbidity, mortality, and a significant burden to health care system with its most profound effects in patients with chronic cardiovascular disease.^1^, ^2^, ^3^ Immunization is an important public health measure through which the cardiovascular sequelae of influenza can be abated.^4^^,^^5^ Adult congenital heart disease (ACHD) patients have significant morbidity leading to an increase in the number of hospital admissions and health care costs.^6^^,^^7^ With advancement of medical and surgical care for this cohort, survival has increased such that today, >90% of infants with CHD survive to adulthood,^8^^,^^9^ and the ACHD population now outnumbers the pediatric CHD population.^10^ In addition, adults admitted with heart failure (HF) have a higher mortality and rehospitalization rate if they have a history of CHD compared to those without a history of CHD.^11^ Previous data have demonstrated that immunization rates in ACHD are lower than the non-ACHD population.^12^^,^^13^ Therefore, any effort to curb influenza-related morbidity and mortality in this growing population may have significant implications.

In addition to immunizing, offering higher dose vaccine to those with reduced antibody-mediated responses is another public health strategy to potentially reduce cardiovascular sequelae of influenza. Current eligible patients for high-dose vaccination include adults 65 years and older. High-dose trivalent versus standard-dose quadrivalent vaccination was further explored in the INVESTED (INfluenza Vaccine to Effectively Stop cardioThoracic Events and Decompensated heart failure trial.^14^ This was a multicenter, double-blind, active comparator-controlled randomized clinical trial conducted in 5,260 patients with high-risk cardiovascular disease in which high-dose trivalent inactivated influenza vaccine (HD-IIV3) did not significantly reduce all-cause mortality or cardiopulmonary hospitalizations compared with standard-dose quadrivalent inactivated influenza vaccine (SD-IIV4).^14^

We hypothesized that self-identified ACHD patients represent a high-risk group for the primary endpoint in the INVESTED trial, namely all-cause death, or cardiopulmonary hospitalization. We therefore performed a prespecified subgroup analysis of the INVESTED trial to describe the patient characteristics, clinical events, adverse events, and potential interaction with randomized treatment arms comparing patients with or without ACHD separated into those receiving HD-IIV3 or SD-IIV4. In addition, we compared ACHD status to other traditional risk factors in predicting all-cause mortality or cardiopulmonary hospitalizations.

## Methods

### Study design

The methodology, design, and protocol of the trial have been summarized previously.^14^^,^^15^ The INVESTED trial was a randomized, multicenter, double-blind, active comparator trial carried out at 157 sites in Canada and the United States. Patients were entered into the study from mid-September until December 31 during the 2016 to 2017 season and until January 31 for the 2017 to 2018 and 2018 to 2019 seasons. Randomization was performed using 1:1 ratio of permuted blocks of random block size ranging from 4 to 6 to maintain double blinding. Vaccines were given intramuscularly, with randomization to HD-IIV3 (containing 60 μg of hemagglutinin per strain) or SD-IIV4 (containing 15 μg of hemagglutinin per strain). Randomization was managed centrally and completed electronically, balanced by center with Stars software (Frontier Science Foundation). The trial was conducted over 3 influenza seasons. Participants received vaccination each year for up to 3 years as per their randomized group allocation. If a participant wanted to withdraw from the trial, they were censored on July 31 of the last season in which they participated, and follow-up was ceased. Trial patients were contacted by phone 1 week after vaccination to elicit adverse reactions to the vaccine and again in the spring and summer.

### Study population

The study population consisted of those hospitalized for acute myocardial infarction (MI) in the prior 12 months or for HF exacerbation in the prior 24 months in addition, to at least one additional risk factor among: older than 65 years, current or prior left ventricular ejection fraction (LVEF) <40%, diabetes mellitus, body mass index ≥30 kg/m^2^, history of chronic kidney disease (defined as estimated glomerular filtration rate ≤60 mL/min for at least 2 readings in the past year), ischemic stroke, peripheral artery disease, current tobacco use, or a MI or HF hospitalization prior to the index hospitalization. ACHD status was self-reported with the answer yes or no to the question: “is there a history of congenital heart disease?” Exclusion criteria included: allergy, hypersensitivity (anaphylaxis), or Guillain-Barre syndrome from influenza vaccine; severe allergy to egg protein; life expectancy <9 months; previous influenza vaccination during enrolling season; acute infection requiring use of antibiotics within 14 days of randomization; known fever within 7 days prior to randomization; pregnancy; or lactation.

### Trial outcomes

The primary outcome of the study was time to a composite of all-cause death or hospitalization for cardiovascular or pulmonary causes. This was examined during each influenza season, with censoring in the first 2 weeks after vaccine administrations and after July 31 of each influenza season. Secondary outcomes were the time to first occurrence of death due to cardiovascular causes or cardiovascular hospitalization within each influenza season, total (first and recurrent) hospitalizations for cardiovascular or pulmonary causes or all-cause death across all influenza seasons, the time to first occurrence of individual components of the primary efficacy endpoint during each influenza season, and the time to first occurrence of all-cause death or hospitalization due to cardiovascular or pulmonary causes across all influenza seasons. The primary safety outcome was vaccine-related adverse events which were assessed 1 week after vaccination. The secondary safety outcome was serious adverse events which included Guillain-Barre syndrome, optic neuritis, Stevens-Johnson syndrome, Bell palsy, encephalitis/myelitis, and toxic epidermal necrolysis. Each outcome was analyzed according to ACHD status, and ACHD status as a risk factor for the primary outcome was also compared in univariate and multivariate analysis.

### Statistical methods

The statistical analysis of the primary outcomes included only those who received vaccinations in any given season, and events of interest were accrued from 14 days after vaccination until July 31 of each enrolling season. Participants without an event of interest were censored on July 31 of each season. Demographic and medical characteristics of the ACHD and non-ACHD groups was summarized with descriptive statistics such as mean ± SD or median (IQR) for quantitative outcomes and frequency (percentage) for qualitative outcomes. Clinical outcomes were compared between those who have a diagnosis of ACHD and those who do not (non-ACHD) using log-rank tests. The analysis of the primary outcome of all-cause death or cardiopulmonary hospitalization was based on a log-rank test with robust variance estimate to account for within participant correlation across multiple seasons, stratified by enrolling season; the corresponding HR and 95% CI were estimated using an unadjusted Cox proportional hazards model, stratified by enrolling season, with robust variance estimate.^16^ ACHD status by treatment interaction was assessed in a similar model as above with the ACHD status by treatment interaction term as an additional covariate in the model. The number of events of all-cause mortality (without hospitalization) and recurrent cardiopulmonary hospitalizations (before all-cause death) was analyzed using a proportional means model.^17^ Statistical analyses was completed using both SAS (SAS Corporation) and R (The R Foundation).

## Results

### Baseline characteristics

During 3 enrolling seasons from September 21, 2016, through January 31, 2019, a total of 5,260 participants were enrolled, 272 self-identified as having ACHD and 4,988 patients not known to have ACHD, who were randomly assigned to receive either high-dose trivalent (n = 132) or standard-dose quadrivalent (n = 140) influenza vaccine. Compared to those without ACHD, the self-identified ACHD cohort was younger, more likely to be women, to be smokers, to have a qualifying event of HF, and have atrial fibrillation as summarized in Table 1 and Central Illustration A. Prior influenza and pneumococcal vaccination rates were similar in both groups.

**Table 1:** Baseline Characteristics of Patients With and Without Congenital Heart Disease

	ACHD Patient No. (%)			Non-ACHD Patient		
	Total(n = 272)^a^	HD-IIV3(n = 132)	SD-IIV4(n = 140)	Total(n = 4,988)^b^	HD-IIV3(n = 2,498)	SD-IIV4(n = 2,490)
Age, y	59.3 ± 14.7	59.9 ± 14.2	58.7 ± 15.2	65.9 ± 12.4	65.8 ± 12.5	65.9 ± 12.3
Sex						
Female	109 (40.1)	48 (36.4)	61 (43.6)	1,364 (27.4)	669 (26.9)	695 (28.0)
Male	163 (59.9)	84 (63.6)	79 (56.4)	3,610 (72.5)	1,820 (73.1)	1,790 (72.0)
Race						
White	208 (76.5)	97 (73.5)	111 (79.3)	3,895 (78.3)	1,945 (78.1)	1,950 (78.4)
Non-White	62 (22.8)	34 (25.8)	28 (20.0)	977 (19.6)	496 (19.9)	481 (19.3)
Qualifying event						
Myocardial infarction	49 (18.0)	25 (18.9)	24 (17.1)	1,911 (38.4)	957 (38.4)	954 (38.4)
Heart failure	223 (82.0)	107 (81.1)	116 (82.9)	3,066 (61.6)	1,534 (61.6)	1,532 (61.6)
Eligibility risk factors						
Prior MI	30 (11.0)	15 (11.4)	15 (10.7)	715 (14.4)	356 (14.3)	359 (14.4)
Prior HF hospitalization	68 (25.0)	31 (23.5)	37 (26.4)	835 (16.8)	419 (16.8)	416 (16.7)
Age ≥65 y	109 (40.1)	54 (40.9)	55 (39.3)	2,878 (57.8)	1,413 (56.7)	1,465 (58.9)
Current or historical LVEF <40%	105 (38.6)	49 (37.1)	56 (40.0)	2,103 (42.3)	1,042 (41.8)	1,061 (42.7)
Type I or type II diabetes mellitus	84 (30.9)	41 (31.1)	43 (30.7)	1,866 (37.5)	935 (37.5)	931 (37.4)
Current BMI ≥30 kg/m^2^	125 (46.0)	57 (43.2)	68 (48.6)	2,426 (48.7)	1,213 (48.7)	1,213 (48.8)
History of renal impairment	92 (33.8)	49 (37.1)	43 (30.7)	1,495 (30.0)	743 (29.8)	752 (30.2)
Current tobacco smoker	67 (24.6)	30 (22.7)	37 (26.4)	835 (16.8)	453 (18.2)	382 (15.4)
History of peripheral artery disease	8 (2.9)	3 (2.3)	5 (3.6)	224 (4.5)	116 (4.7)	108 (4.3)
History of ischemic stroke	53 (19.5)	30 (22.7)	23 (16.4)	380 (7.6)	176 (7.1)	204 (8.2)
Number of risk factors						
1	63 (23.2)	32 (24.2)	31 (22.1)	1,013 (20.4)	505 (20.3)	508 (20.4)
2	73 (26.8)	34 (25.8)	39 (27.9)	1,350 (27.1)	703 (28.2)	647 (26.0)
3	60 (22.1)	27 (20.5)	33 (23.6)	1,235 (24.8)	589 (23.6)	646 (26.0)
4 or more	76 (27.9)	39 (29.5)	37 (26.4)	1,379 (27.7)	694 (27.9)	685 (27.6)
Other medical/surgical history						
Hypertension	198 (72.8)	93 (70.5)	105 (75.0)	3,848 (77.3)	1,893 (76.0)	1,955 (78.6)
Dyslipidemia	151 (55.5)	73 (55.3)	78 (55.7)	3,465 (69.6)	1,720 (69.0)	1,745 (70.2)
Asthma	63 (23.2)	29 (22.0)	34 (24.3)	539 (10.8)	279 (11.2)	260 (10.5)
Chronic obstructive pulmonary disease	59 (21.7)	28 (21.2)	31 (22.1)	947 (19.0)	458 (18.4)	489 (19.7)
Percutaneous coronary intervention	71 (26.1)	38 (28.8)	33 (23.6)	2,091 (42.0)	1,065 (42.8)	1,026 (41.3)
Coronary artery bypass graft	56 (20.6)	28 (21.2)	28 (20.0)	984 (19.8)	475 (19.1)	509 (20.5)
Atrial fibrillation	120 (44.1)	62 (47.0)	58 (41.4)	1,605 (32.2)	792 (31.8)	813 (32.7)
ICD	65 (23.9)	31 (23.5)	34 (24.3)	891 (17.9)	432 (17.3)	459 (18.5)

Values are mean ± SD or n (%).

ACHD = adult congenital heart disease; BMI = body mass index; HD-IIV3 = high-dose trivalent inactivated influenza vaccine; HF = heart failure; LVEF = left ventricular ejection fraction; MI = myocardial infarction; SD-IIV4 = standard-dose quadrivalent inactivated influenza vaccine; SD = standard deviation.

a Represents all randomized participants.

b Unique participant numbers of 2,474 in the high-dose group and 2,465 in the low-dose group add up to 4,939, rather than the 4,988.

**Central Illustration:**
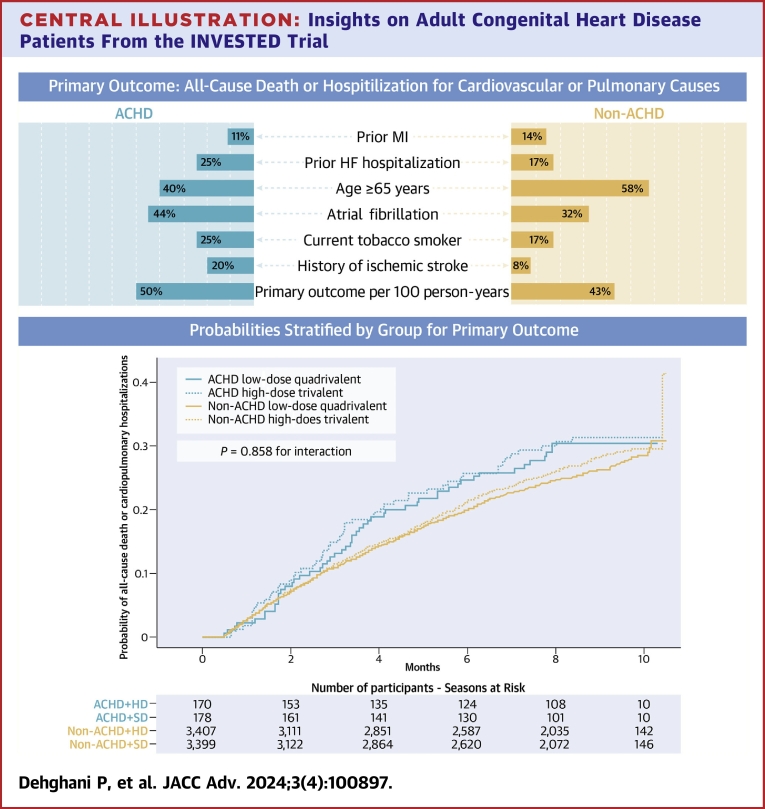
Insights on Adult Congenital Heart Disease Patients From the INVESTED Trial Distinct differences exist in the clinical demographics between ACHD patients and non-ACHD patients, including the prevalence of all cause death or hospitalization for cardiovascular or pulmonary causes. Probabilities stratified by groups for the primary outcome. The interaction was at *P* = 0.858. ACHD = adult congenital heart disease; HF = heart failure; INVESTED = INfluenza Vaccine to Effectively Stop cardioThoracic Events and Decompensated heart failure; MI = myocardial infarction.

### Outcomes according to ACHD status

**Figure 1:**
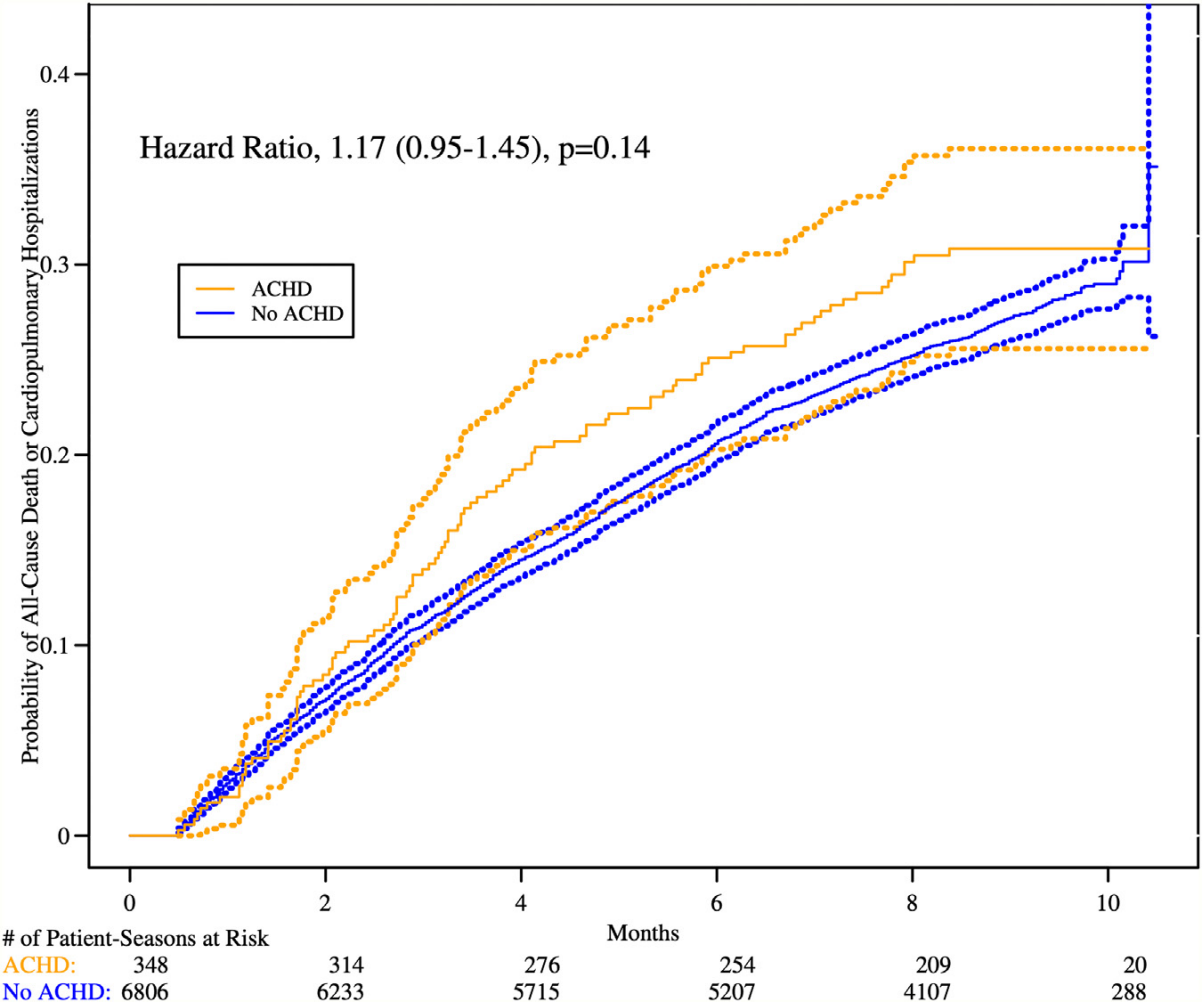
**Primary Outcome According to Congenital Heart Disease Status** ACHD = adult congenital heart disease.

The median duration of follow-up was 9 months for the non-ACHD group and 9.07 months in the self-identified ACHD group. The primary outcome was similar in those with compared to those without ACHD: 49.8 versus 42.8 per 100 person-years with an adjusted hazard ratio (aHR) of 1.17 (95% CI: 0.95-1.45; *P* = 0.14) (Table 2, Figure 1). All-cause death rates were also similar: 4.8 versus 3.8 per 100 person-years (aHR: 1.25; 95% CI: 0.66-2.37. Hospitalizations for cardiovascular or pulmonary causes were also similar: 45.0 versus 39.0 per 100 person-years, with aHR of 1.15 (95% CI: 0.94-1.42) (Table 2, Figure 1).

**Table 2:** Outcomes According to Congenital Heart Disease Status

	ACHD		No ACHD		RateDiff.	Difference 95% CI	Hazard/Mean Ratio	95% CI	*P* Value
	Total	Rate	Total	Rate					
Primary outcome (mITT)	(n = 348)		(n = 6,806)						
All-cause death or cardiopulmonary hospitalization^a^	104	0.498	1,795	0.428	0.069	(−0.022 to 0.161)	1.173	(0.947-1.453)	0.144
Year 1	6	0.610	169	0.617	−0.006	(−0.506 to 0.493)	0.993	(0.456-2.162)	0.985
Year 2	47	0.578	743	0.466	0.112	(−0.041 to 0.265)	1.240	(0.920-1.671)	0.158
Year 3	51	0.433	883	0.380	0.053	(−0.062 to 0.167)	1.141	(0.857-1.519)	0.365
All-cause death (without hospitalizations)	10	0.048	160	0.038					
Year 1	0	0.000	16	0.058					
Year 2	6	0.074	61	0.038					
Year 3	4	0.034	83	0.036					
Cardiopulmonary hospitalizations (before all-cause death)	94	0.450	1,635	0.390					
Year 1	6	0.610	153	0.558					
Year 2	41	0.505	682	0.428					
Year 3	47	0.399	800	0.344					

ACHD = adult congenital heart disease; ICD = implantable cardioverter-defibrillator; mITT = modified intention-to-treat.

a Based on Cox proportional hazards model with robust variance estimate.

### Outcomes according to treatment group

Among patients with ACHD, there were 52 primary events (47 hospitalizations for cardiovascular or pulmonary causes and 5 deaths from any cause) in 132 unique participants (event rate 50.5 per 100 patient-years) among 170 participant-seasons in the high-dose group compared with 52 primary events (47 hospitalizations for cardiovascular or pulmonary causes and 5 deaths from any cause) in 139 unique participants (event rate 49.1 per 100 patients-years) among 178 participant-seasons in the standard-dose group (HR: 1.034; 95% CI: 0.687-1.555; *P* = 0.87). Among patients without ACHD, there were 923 primary events (836 hospitalizations for cardiovascular or pulmonary causes and 87 deaths from any cause) in 2,474 unique participants (event rate 44.2 per 100 patient-years) among 3,407 participant-seasons in the high-dose group compared with 872 primary events (799 hospitalizations for cardiovascular or pulmonary causes and 73 deaths from any cause) in 2,465 unique participants (event rate 41.5 per 100 patients-years) among 3,399 participant-seasons in the standard dose group (HR: 1.06; 95% CI: (0.965-1.177); *P* = 0.21). The *P* value for interaction between ACHD status and randomized treatment effect for the primary outcome was not significant with *P* = 0.858 (Table 3, Central Illustration B).

**Table 3:** Primary and Secondary Outcomes in a Study of the Effect of High-Dose vs Standard-Dose Influenza Vaccine According to ACHD Status

	ACHD				Hazard/Mean Ratio	*P* Value	No ACHD				Hazard/Mean Ratio	*P* Value	*P* Value for Interaction
	HD-IIV3		SD-IIV4				HD-IIV3		SD-IIV4				
	Total	Rate	Total	Rate			Total	Rate	Total	Rate			
Primary outcome (mITT)	(n = 170)		(n = 178)				(n = 3,407)		(n = 3,399)				
All-cause death or cardiopulmonary hospitalization^a^	52	0.505	52	0.491	1.034	0.874	923	0.442	872	0.415	1.066	0.209	0.858
Year 1	1	0.211	5	0.984	0.221	0.176	90	0.655	79	0.578	1.132	0.421	0.136
Year 2	27	0.645	20	0.508	1.278	0.402	386	0.492	357	0.441	1.115	0.140	0.665
Year 3	24	0.426	27	0.439	0.988	0.965	447	0.383	436	0.377	1.014	0.832	0.881
All-cause death (without hospitalizations)	5	0.049	5	0.047			87	0.042	73	0.035			
Year 1	0	0.000	0	0.000			11	0.080	5	0.037			
Year 2	4	0.095	2	0.051			32	0.041	29	0.036			
Year 3	1	0.018	3	0.049			44	0.038	39	0.034			
Cardiopulmonary hospitalizations (before all-cause death)	47	0.456	47	0.444			836	0.400	799	0.380			
Year 1	1	0.211	5	0.984			79	0.575	74	0.541			
Year 2	23	0.549	18	0.457			354	0.452	328	0.406			
Year 3	23	0.408	24	0.390			403	0.345	397	0.344			

ACHD = adult congenital heart disease; HD-IIV3 = high-dose trivalent inactivated influenza vaccine; SD-IIV4 = standard-dose quadrivalent inactivated influenza vaccine.

a Based on Cox proportional hazards model with robust variance estimate.

### Adverse events

Overall, influenza vaccinations were well tolerated and there were no notable differences between self-identified ACHD and non-ACHD groups in the high-dose group (see Table 4). In the standard-dose group, 6 of 139 self-identified ACHD patients (4.3%) versus 38 of 2,465 of non-ACHD patients (1.5%) had severe vaccine-related adverse events.

**Table 4:** Postvaccination Adverse Events (Within 1 Week) in a Study of the Effect of High-Dose vs Standard-Dose Influenza Vaccine According to ACHD Status

	HD-IIV3		SD-IIV4	
	ACHD	No ACHD	ACHD	No ACHD
	(132 part. in 170 part.-seasons)	(2,474 part. in 3,407 part.-seasons)	(139 part. n 178 part.-seasons)	(2,465 part. in 3,399 part.-seasons)
Pain	46 (27.1)	886 (26.0)	37 (20.8)	646 (19.0)
Myalgia	25 (14.7)	475 (13.9)	29 (16.3)	394 (11.6)
Overall discomfort	15 (8.8)	295 (8.7)	17 (9.6)	263 (7.7)
Headache	15 (8.8)	264 (7.7)	21 (11.8)	251 (7.4)
Swelling	7 (4.1)	191 (5.6)	8 (4.5)	111 (3.3)
Erythema	7 (4.1)	150 (4.4)	7 (3.9)	150 (4.4)
Fever	8 (4.7)	94 (2.8)	8 (4.5)	70 (2.1)
Any of above	74 (43.5)	1,375 (40.4)	70 (39.3)	1,159 (34.1)
Severity (by vaccination)				
None	95 (55.9)	1,975 (58.0)	105 (59.0)	2,183 (64.2)
Mild	61 (35.9)	1,047 (30.7)	56 (31.5)	933 (27.4)
Moderate	12 (7.1)	331 (9.7)	11 (6.2)	244 (7.2)
Severe	2 (1.2)	54 (1.6)	6 (3.4)	39 (1.1)
Severity (by participant)				
None	72 (54.5)	1,296 (52.4)	74 (53.2)	1,439 (58.4)
Mild	47 (35.6)	817 (33.0)	49 (35.3)	761 (30.9)
Moderate	11 (8.3)	308 (12.4)	10 (7.2)	227 (9.2)
Severe	2 (1.5)	53 (2.1)	6 (4.3)	38 (1.5)
Participants with any vaccine-related adverse event	60 (45.5)	1,181 (47.7)	65 (46.8)	1,031 (41.8)
Participants with any severe vaccine-related adverse event	2 (1.5)	53 (2.1)	6 (4.3)	38 (1.5)

ACHD = adult congenital heart disease; HD-IIV3 = high-dose trivalent inactivated influenza vaccine; SD-IIV4 = standard-dose quadrivalent inactivated influenza vaccine.

### Magnitude of risk according to congenital heart disease status

**Figure 2:**
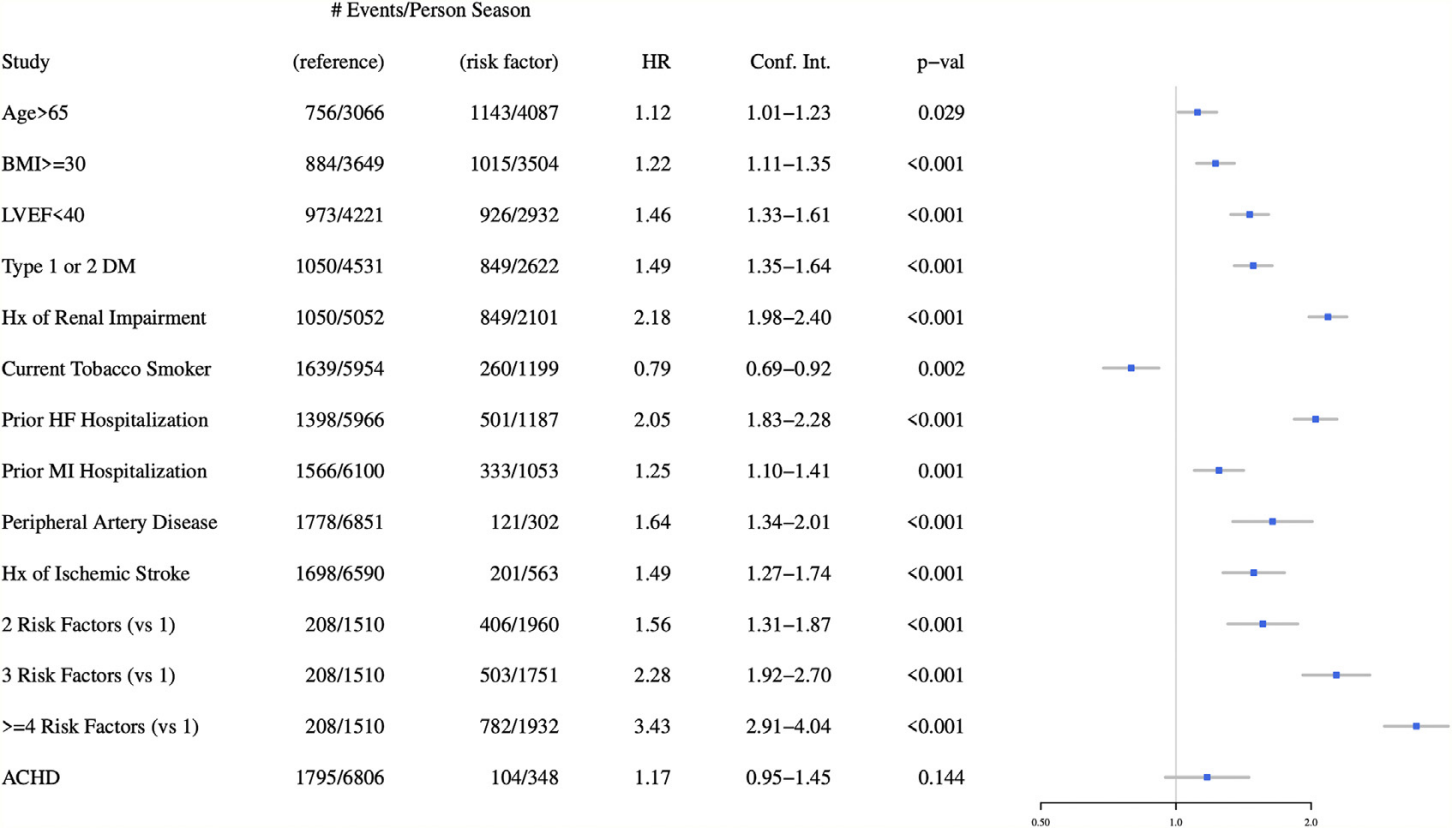
**Selected Baseline Characteristics as Risk Factors for Primary Outcome in Univariate Analysis** ACHD = adult congenital heart disease; BMI = body mass index; DM = diabetes mellitus; HF = heart failure; Hx = history; LVEF = left ventricular ejection fraction; MI = myocardial infarction.

**Figure 3:**
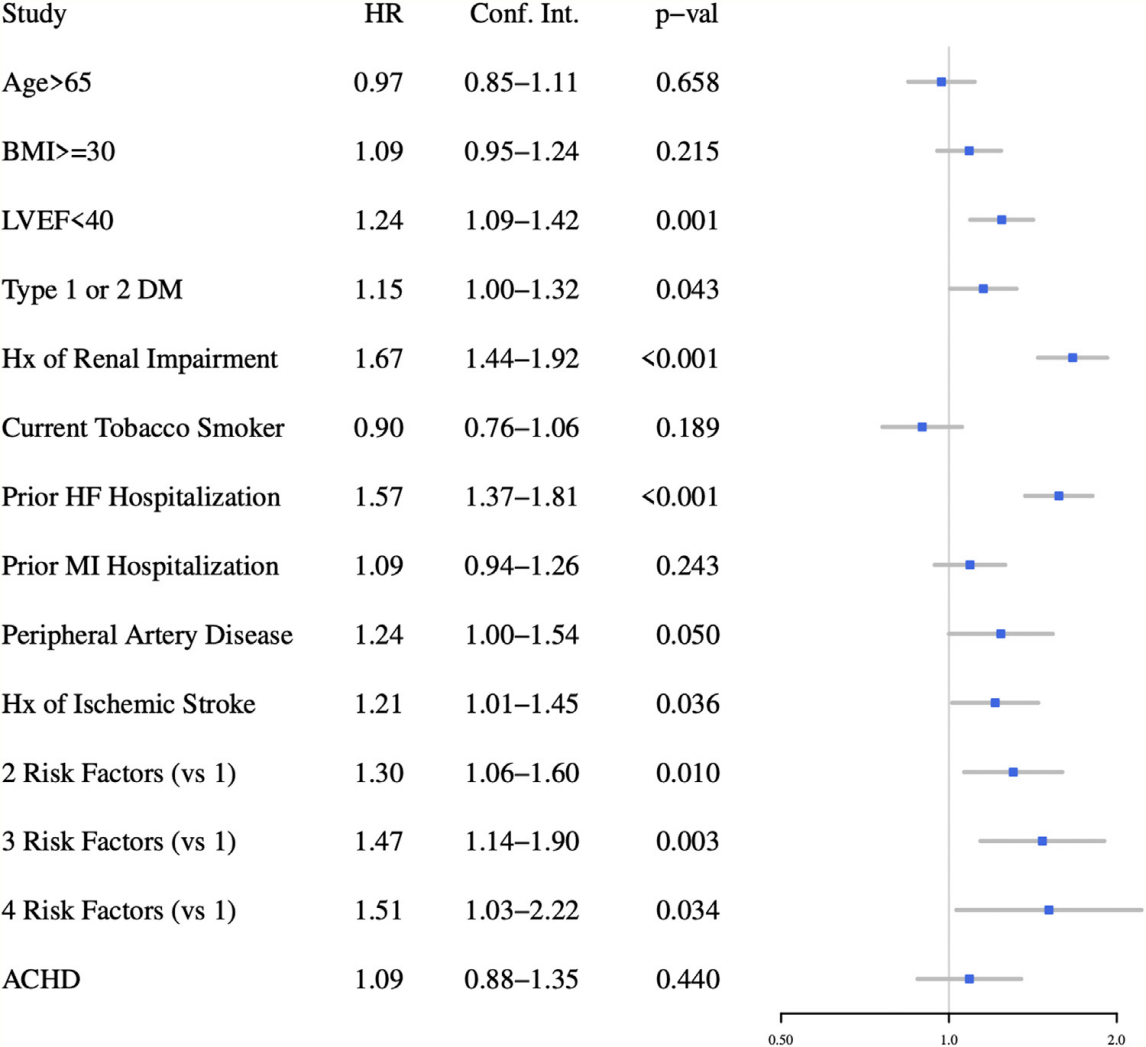
**Selected Baseline Characteristics as Risk Factors for Primary Outcome in Multivariate Analysis** ACHD = adult congenital heart disease; BMI= body mass index; DM = diabetes mellitus; HF = heart failure; Hx = history; LVEF = left ventricular ejection fraction; MI = myocardial infarction.

ACHD was also examined through univariate and multivariate analysis in comparison to other risk factors for predicting the primary outcome of all-cause mortality or cardiopulmonary hospitalization (Figures 2 and 3). ACHD status was not associated with the primary outcome in either univariate (HR: 1.17; 95% CI: 0.95-1.45; *P* = 0.14 or multivariate (aHR: 1.09; 95% CI: 0.88-1.35; *P* = 0.44) analysis.

## Discussion

We report several important findings from this prespecified subgroup analysis of self-identified ACHD patients in the INVESTED trial. Firstly, self-identified ACHD patients had a different risk factor profile than those without ACHD enrolled in the INVESTED trial. Secondly, the rates of all-cause mortality and/or cardiopulmonary hospitalization were similar in self-identified ACHD patients compared to non-ACHD patients. Finally, despite a similar HR to other well established risk factors, ACHD status did not alter the INVESTED trials’ overall lack of different effect on the primary outcome in high-dose compared with low-dose influenza vaccination.

Our self-identified ACHD cohort had a different risk profile compared to non-ACHD patients in INVESTED trial and other landmark trials. More self-identified ACHD patients were active smokers compared to non-ACHD patients; 25% versus 15% to 18% in INVESTED and other landmark trials.^18^^,^^19^ In addition, 44.1% of self-identified ACHD patients had atrial fibrillation compared to 32% of non-ACHD patients in INVESTED, and 36% to 39% in other landmark HF trials.^20^, ^21^, ^22^ Stroke rate was also significantly higher: 19.5% of ACHD patients had a previous history of stroke compared to 7% in the non-ACHD group in INVESTED, and 7% to 9% in other trials.^19^^,^^20^ Bouchardy et al^23^ found that the presence of an atrial arrhythmia in ACHD increased the risk of stroke and death by close to 50%. Lanz et al^24^ found the prevalence of stroke in ACHD to be 1 in 11 for men and 1 in 15 for women, and the 30-day mortality after ischemic stroke in ACHD was 5.1%. Despite the self-identified ACHD patients being younger by an average of 6 years, prevalence of renal impairment and hypertension was similar to the non-ACHD cohort. While this may reflect INVESTED trial’s inclusion criteria of a higher risk patient population, it could also speak to altered vascular hemodynamics in CHD such as those with coarctation or Fontan physiology. The higher-than-expected smoking rate as seen in our ACHD cohort has been previously described in previous ACHD reports^25^ and may also explain the higher rates of chronic obstructive pulmonary disease (COPD) in our cohort.

Why is it that despite having higher rates of atrial fibrillation, stroke, and smoking status, our self-identified ACHD cohort had similar risk for death and cardiopulmonary hospitalization, a finding that is contrary to the literature^11^? This may be because our ACHD cohorts were younger, and more likely to be women, essentially counterbalancing overall risk. Our ACHD cohort may also have had a disproportionally higher rate of mild anatomical complexity and/or less advanced physiologic staging, a hypothesis that cannot be confirmed due to limitation of our current data set. Similar outcomes between the ACHD and non-ACHD cohorts can also be explained by the overall cardioprotective properties of the influenza vaccine offered to everyone in this trial. Although low uptake of influenza vaccination in ACHD patients is well-documented,^12^^,^^13^ this was not observed in our study—likely a reflection of selection bias, as patients interested and willing to enroll in a vaccination trial may have greater knowledge of influenza vaccination as compared with the general public. Finally, our median duration follow-up of 9 months may have been inadequate. Contemporary data suggest that CHD-related mortality no longer occurs at the time of surgical intervention,^26^^,^^27^ but rather, later in life. A median duration follow-up of 9 years was required in a 7,000 ACHD patients British registry to demonstrate a mortality rate of 7.7%.^26^

Despite having a similar LVEF as those without ACHD patients, self-identified ACHD patients were more likely to have a history of HF hospitalization (25% vs 17%), and their index event was more likely to be congestive HF (82% vs 62%). This presumably relates to a lower risk for the other possible reason for inclusion, being MI, in the younger self-identified ACHD subgroup. Given a higher anticipated prevalence of right ventricular causes of “HF” in the ACHD cohort, it may be surprising that the LVEF was similar in the ACHD and non-ACHD cohorts. This may be explained by inadequate data collection on systemic ventricular dysfunction in patients with morphologic right ventricles. Even though ACHD patients are living longer, and their comorbidities and HF admissions are increasing,^28^ relying solely on LVEF may be misleading.^26^ Causes of HF in ACHD patients are multifactorial including volume and pressure overload from residual lesions, excessive neurohormonal activation, and an increased rate of atrial tachyarrhythmias.^29^, ^30^, ^31^ For instance, in some patients with transposition of the great arteries (TGA), the systemic ventricle has right ventricular physiology, therefore less robust in its response to volume and pressure overload.^29^ Similarly, patients with Fontan circulation are at high risk of HF with moderate to severe systolic dysfunction described only in one third of such patients.^29^^,^^32^ Additionally, ACHD is associated with excessive neurohormonal activation, thought to be secondary to abnormal cardiac myocyte distension, with increased activation of this system likely contributing to cardiac deterioration.^29^^,^^33^ Regardless of the mechanism, HF is a major cause of mortality in the ACHD cohort, described in up to 45% of deaths in ACHD registries.^26^, ^27^, ^28^^,^^34^

Unfortunately, in addition to complex pathophysiology that precludes uniform approaches to HF management in ACHD, there is also a paucity of evidence to guide management in this population. ACHD are part of a larger underrepresented group in clinical trials of HF, including racial and ethnic minorities who share the common trait of higher rates of HF but have been least studied in clinical trials.^35^ For example, landmark clinical trials of HF which form the evidence for optimal goal-directed medical therapy, such as the RALES (Randomized Aldactone Evaluation Study) and SHIFT (Systolic Heart failure treatment with the *I*_f_ inhibitor ivabradine Trial) specifically excluded ACHD.^19^^,^^36^ Our trial was able to successfully include a prespecified subgroup analysis of ACHD patients, demonstrating that this is a population that can be effectively and safely included in clinical trials.

Despite a different profile of risk, high-dose compared with low-dose influenza vaccination did not reduce the primary outcome in ACHD patients. One explanation is that our trial did not examine rates of influenza infection, which high-dose vaccination may have reduced based on previous data.^37^^,^^38^ Additionally, this trial compared high-dose trivalent vaccine to standard-dose quadrivalent vaccine; the quadrivalent vaccine used for the standard-dose group contains an additional B/Yamagata strain which could have attenuated differences that would have been seen due to vaccine dosage.^16^ Although this trial did not demonstrate a difference between the 2 vaccine formulations in ACHD, we want to emphasize that this does not preclude the benefit of vaccination compared to the unvaccinated. The Centers for Disease Control and Prevention (CDC) recommends annual influenza vaccination in those aged 6 months and above, which also holds true for ACHD patients.^39^

### Study Limitations

This study and associated subgroup analysis has several limitations. Most importantly, our study did not include granular information on anatomical and/or physiologic classification of their underlying congenital lesions. Adults with CHD represent a heterogenous group of patients, with clear incremental prognostic implications depending on degree of anatomic complexity and/or physiologic staging.^31^^,^^40^^,^^41^ Additionally, ACHD status was self-determined and not externally validated. Although incorrect classification is possible, it is plausible that adult self-reporting was accurate in our series as the ACHD cohort were younger, and had a higher prevalence of stroke and atrial fibrillation, characteristics that are well established in ACHD patient population.^42^ Furthermore, this subgroup analysis was performed on a trial with neutral results that was stopped early because the required event rate was surpassed. While our 5% prevalence of self-reported ACHD is higher than the accepted 0.8 to 1% prevalence of births with CHD,^43^ the INVESTED population was already enriched with cardiac comorbidities with an admission related to HF or MI. A 7% prevalence of MI in ACHD has been documented,^44^ which is considered higher than that of the general population. Lastly, influenza infection rates were not assessed, as the study was structured as a pragmatic trial examining clinical outcomes.^14^ Therefore, we cannot exclude the possibility that high-dose vaccine could reduce the rate of influenza vaccination in ACHD.^14^^,^^45^

## Conclusions

While self-identified ACHD patients had a different profile of risk and comorbidities compared to those without ACHD, they had similar primary outcome of all-cause mortality or cardiopulmonary hospitalization. Similar to the original INVESTED trial, our sub-study of self-identified ACHD patients did not show a difference between high-dose and low-dose influenza vaccination for the primary outcome. Our experience demonstrates that including ACHD patients within larger cardiovascular trials is safe and feasible. We strongly encourage strategies to enroll ACHD patients in future landmark cardiovascular trials, particularly those focusing on HF.

PERSPECTIVES **COMPETENCY IN MEDICAL KNOWLEDGE:** Despite similar rates of all-cause mortality or cardiopulmonary hospitalization, self-identified ACHD patients have different demographic and clinic risk factors compared to non-ACHD patients. Furthermore, these rates did not differ in those receiving high-dose trivalent or standard-dose quadrivalent influenza vaccine.

**TRANSLATIONAL OUTLOOK:** Further research is required to understand how the anatomical and/or physiologic classification of these patients’ underlying congenital lesions influence clinical outcome of ACHD patients.

## Funding support and author disclosures

Internal funding was provided by Prairie Vascular Research Inc. Dr Goodman has received research grant support (eg, steering committee or data and safety monitoring committee) and/or speaker/consulting honoraria (eg, advisory boards) from Amgen, Anthos Therapeutics, AstraZeneca, Bayer, Boehringer Ingelheim, Bristol Myers Squibb, CSL Behring, Daiichi-Sankyo/American Regent, Eli Lilly, Esperion, Ferring Pharmaceuticals, HLS Therapeutics, JAMP Pharma, Merck, Novartis, Novo Nordisk A/C, Pendopharm/Pharmascience, Pfizer, Regeneron, Sanofi, Servier, Tolmar Pharmaceuticals, Valeo Pharma; and salary support/honoraria from the Heart and Stroke Foundation of Ontario/University of Toronto (Polo) Chair, Canadian Heart Failure Society, Canadian Heart Research Centre and MD Primer, Canadian VIGOUR Centre, Cleveland Clinic Coordinating Centre for Clinical Research, Duke Clinical Research Institute, New York University Clinical Coordinating Centre, PERFUSE Research Institute, TIMI Study Group (Brigham Health). Dr Hegde has received fees paid to institution from Myokardia. Dr Bhatt discloses the following relationships - Advisory Board for Angiowave, Bayer, Boehringer Ingelheim, Cardax, CellProthera, Cereno Scientific, Elsevier Practice Update Cardiology, High Enroll, Janssen, Level Ex, McKinsey, Medscape Cardiology, Merck, MyoKardia, NirvaMed, Novo Nordisk, PhaseBio, PLx Pharma, Regado Biosciences, Stasys; Board of Directors; Angiowave (stock options), Boston VA Research Institute, Bristol Myers Squibb (stock), DRS.LINQ (stock options), High Enroll (stock), Society of Cardiovascular Patient Care, TobeSoft; Chair: Inaugural Chair; American Heart Association Quality Oversight Committee; Consultant: Broadview Ventures; Data Monitoring Committees: Acesion Pharma, Assistance Publique-Hôpitaux de Paris, Baim Institute for Clinical Research (formerly Harvard Clinical Research Institute, for the PORTICO trial, funded by St. Jude Medical, now Abbott), Boston Scientific (Chair, PEITHO trial), Cleveland Clinic (including for the ExCEED trial, funded by Edwards), Contego Medical (Chair, PERFORMANCE 2), Duke Clinical Research Institute, Mayo Clinic, Mount Sinai School of Medicine (for the ENVISAGE trial, funded by Daiichi Sankyo; for the ABILITY-DM trial, funded by Concept Medical), Novartis, Population Health Research Institute; Rutgers University (for the NIH-funded MINT Trial); Honoraria; American College of Cardiology (Senior Associate Editor, Clinical Trials and News, ACC.org; Chair, ACC Accreditation Oversight Committee), Arnold and Porter law firm (work related to Sanofi/Bristol-Myers Squibb clopidogrel litigation), Baim Institute for Clinical Research (formerly Harvard Clinical Research Institute; RE-DUAL PCI clinical trial steering committee funded by Boehringer Ingelheim; AEGIS-II executive committee funded by CSL Behring), Belvoir Publications (Editor in Chief, Harvard Heart Letter), Canadian Medical and Surgical Knowledge Translation Research Group (clinical trial steering committees), Cowen and Company, Duke Clinical Research Institute (clinical trial steering committees, including for the PRONOUNCE trial, funded by Ferring Pharmaceuticals), HMP Global (Editor in Chief, Journal of Invasive Cardiology), Journal of the American College of Cardiology (Guest Editor; Associate Editor), K2P (Co-Chair, interdisciplinary curriculum), Level Ex, Medtelligence/ReachMD (CME steering committees), MJH Life Sciences, Oakstone CME (Course Director, Comprehensive Review of Interventional Cardiology), Piper Sandler, Population Health Research Institute (for the COMPASS operations committee, publications committee, steering committee, and USA national co-leader, funded by Bayer), Slack Publications (Chief Medical Editor, Cardiology Today’s Intervention), Society of Cardiovascular Patient Care (Secretary/Treasurer), WebMD (CME steering committees), Wiley (steering committee); Other: Clinical Cardiology (Deputy Editor), NCDR-ACTION Registry Steering Committee (Chair), VA CART Research and Publications Committee (Chair); Patent: Sotagliflozin (named on a patent for sotagliflozin assigned to Brigham and Women's Hospital who assigned to Lexicon; neither I nor Brigham and Women's Hospital receive any income from this patent); Research Funding: Abbott, Acesion Pharma, Afimmune, Aker Biomarine, Amarin, Amgen, AstraZeneca, Bayer, Beren, Boehringer Ingelheim, Boston Scientific, Bristol-Myers Squibb, Cardax, CellProthera, Cereno Scientific, Chiesi, CinCor, CSL Behring, Eisai, Ethicon, Faraday Pharmaceuticals, Ferring Pharmaceuticals, Forest Laboratories, Fractyl, Garmin, HLS Therapeutics, Idorsia, Ironwood, Ischemix, Janssen, Javelin, Lexicon, Lilly, Medtronic, Merck, Moderna, MyoKardia, NirvaMed, Novartis, Novo Nordisk, Owkin, Pfizer, PhaseBio, PLx Pharma, Recardio, Regeneron, Reid Hoffman Foundation, Roche, Sanofi, Stasys, Synaptic, The Medicines Company, Youngene, 89Bio; Royalties: Elsevier (Editor, Braunwald’s Heart Disease); Site Co-Investigator: Abbott, Biotronik, Boston Scientific, CSI, Endotronix, St. Jude Medical (now Abbott), Philips, SpectraWAVE, Svelte, Vascular Solutions; Trustee: American College of Cardiology; Unfunded Research: FlowCo, Takeda. All other authors have reported that they have no relationships relevant to the contents of this paper to disclose.
